# Use of qualitative behavioural assessment to investigate affective states of housed dairy cows under different environmental conditions

**DOI:** 10.3389/fvets.2023.1099170

**Published:** 2023-03-17

**Authors:** Alison L. Russell, Laura V. Randall, Jasmeet Kaler, Nikki Eyre, Martin J. Green

**Affiliations:** School of Veterinary Medicine and Science, University of Nottingham, Sutton Bonington Campus, Leicestershire, United Kingdom

**Keywords:** qualitative behavioural assessment, cow, affect, positive welfare, enrichment

## Abstract

In addition to the reduction of suboptimal welfare, there is now a need to provide farmed animals with positive opportunities to provide confidence that they have experienced a life worth living. Diversification of the environment through environmental enrichment strategies is one suggested avenue for providing animals with opportunities for positive experiences. The provision of more stimulating environmental conditions has been widely implemented in other animal production industries, based on evidenced welfare benefits. However, the implementation of enrichment on dairy farms is limited. In addition to this, the relationship between enrichment and dairy cows' affective states is an under-researched area. One specific welfare benefit of enrichment strategies which has been observed in a number of species, is increased affective wellbeing. This study investigated whether the provision of different forms of environmental enrichment resources would impact the affective states of housed dairy cows. This was measured by Qualitative Behavioural Assessment, currently a promising positive welfare indicator. Two groups of cows experienced three treatment periods; (i) access to an indoor novel object, (ii) access to an outdoor concrete yard and (iii) simultaneous access to both resources. Principal component analysis was used to analyse qualitative behavioural assessment scores, which yielded two principal components. The first principal component was most positively associated with the terms “content/relaxed/positively occupied” and had the most negative associations with the terms ‘fearful/bored'. A second principal component was most positively associated with the terms “lively/inquisitive/playful” and was most negatively associated with the terms “apathetic/bored”. Treatment period had a significant effect on both principal components, with cows being assessed as more content, relaxed and positively occupied and less fearful and bored, during periods of access to additional environmental resources. Similarly, cows were scored as livelier, more inquisitive and less bored and apathetic, during treatment periods compared to standard housing conditions. Concurrent with research in other species, these results suggest that the provision of additional environmental resources facilitates positive experiences and therefore enhanced affective states for housed dairy cows.

## Introduction

Affective experiences are an inherent component of the overall welfare state of an animal (1, 2). Recently, as research into the emotional experiences of animals has developed, there has been a shift in the focus of animal welfare to advance from simply the reduction of suffering, which avoids poor welfare, to also provide animals with positive experiences (3, 4). There is currently demand for the development and implementation of positive welfare opportunities for farm animals, to ensure that they have experienced an acceptable quality of life (5). This stems from ongoing societal concern regarding the quality of lives of intensively housed livestock (6, 7), including dairy cows (8, 9). Evaluation of animals' affective states is an ongoing complex challenge, with the lack of a gold standard assessment (10, 11). Yet to be able to assess the success of interventions aimed at offering opportunities for positive welfare, evaluation of affective states is imperative.

One avenue that has been suggested to offer confined animals with opportunities for positive experiences is diversification of the environment (11, 12). This may provide opportunities beyond that of solely meeting basic needs, such as the facilitation of exploration, agency or a greater repertoire of behaviours (11, 12). Therefore, enrichment interventions are often implemented with the strategic goal of enhancing animals' affective states. The relationship between animals' living conditions and their affective experiences have started to be explored. Indications of more positive affective states were found following either a period of environmental enrichment or in animals housed in more stimulus diverse compared to basic housing conditions in different species including, dairy cows (13, 14), dairy calves (15, 16), chickens (17, 18), pigs (19, 20) and rats (21, 22). The inverse effect has also been observed in starlings, through the use of judgement bias, which monitors animals' responses to ambiguous situations to infer affective valence (23). Starlings expressed a pessimistic bias indicative of poorer affective states, following removal of enriched conditions (24). Similarly, increased negative behavioural decision-making has been observed in pigs that had previously spent time in enriched housing and were then transferred to barren housing, compared to pigs that had only ever experienced barren housing (19). Crump et al. (25) investigated whether pasture access improved emotional states in dairy cows and reported that cows which had access to pasture approached a known food reward slower than cows that were fully housed. The authors proposed that the explanation was a reduced reward anticipation, generally shown when higher or more frequent rewards are experienced in day-to-day life (26), concluding that pasture access may facilitate more rewarding lives and therefore better welfare. Environmental enrichment has been widely implemented in several other industries (27, 28) based on its contribution to welfare. However, its implementation on dairy farms is limited and the development of enrichment methods specifically for housed cows is required (29).

One method that has emerged for assessment of affective states of animals is Qualitative Behavioural Assessment (QBA). The method uses observer evaluation and interpretation of animals' behavioural expressive demeanour and the nature of their interaction with the environment (30, 31). The qualitative assessment aims to describe the animals' experience within its setting, by evaluating not solely what an animal is doing but by how it behaves, which encapsulates its subjective affective states (31). This information is collated through descriptive terms, which are then used to formulate quantitative variables. QBA is regarded as assessing more than physical body language – it is assumed to assess a psychological dimension of behavioural expressivity allowing judgement to be made of the quality of an animal's experience (31). QBA has been described as one of the most promising positive welfare indicators currently available, based on the breadth of evidence regarding its validity and reliability (10, 32, 33). Alongside this, QBA is very feasible as it requires little time or resources (34, 35), in contrast to other behavioural or physiological positive welfare indicators. This is particularly practical for the assessment of farm animal welfare. QBA is currently the only measure of positive affective state to be practically incorporated into on farm animal welfare assessments in the UK and is currently being used by two independent welfare assurance bodies (36, 37). QBA results have been shown to be concurrent with some physical health indicators in different species (38–40) and other behavioural tests linked to affect (39, 41). This is however not always the case, with other studies finding no correlations between QBA results and physical health indicators (42–44) or wider farm assurance assessment protocols (45). The technique has identified biologically plausible differences in behavioural expression and associated affective states in dairy cows infected with mastitis (38), in both positive and negative social situations (46) and between cows from tethered and loose housing systems (47). The technique has previously been used to directly evaluate the affective states of animals in different housing conditions, with results conducive to enhanced emotional wellbeing, in extensive compared to intensive systems in pigs (48), enriched compared to unenriched housing in pigs (20) and dairy goats with access to pasture compared to without (49).

The purpose of this research was to explore the relationship between housing conditions and affective states of dairy cattle. Our specific aim was to evaluate whether QBA could be used to detect changes in cows' behavioural expression during periods of altered housing conditions, comprising of access to an outdoor exercise area and provision of an indoor novel object.

## Materials and methods

### Ethical approval

The study was granted ethical approval by The University of Nottingham, School of Veterinary Medicine and Science Ethical Review Committee, approval number 2697-190221. All methods were performed in accordance with the relevant guidelines and regulations.

### Animals and housing

**Figure 1:**
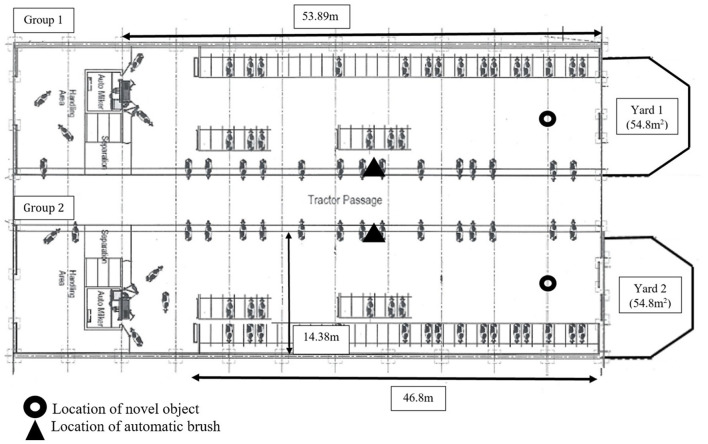
Schematic view of the experimental housing for both groups of cows within the study.

The study was conducted at the Center for Dairy Science Innovation, University of Nottingham, a continually housed 300-cow research dairy herd, producing milk commercially. Cows in the experimental groups were continually housed in two identical 774.9 m^2^ buildings, containing 51 sand-bedded cubicles with concrete slatted flooring, scraped automatically daily (Figure 1). Subjects received ad libitum access to fresh water *via* three water troughs and were fed a total mixed ration (TMR) ad libitum which was replenished daily at 09:00. Subjects were milked robotically *via* a Lely automatic milking system where they received additional concentrate feed. One automatic brush was available in each building.

We selected 96 cows and assigned them to two separate study groups to repeat one experimental trial. Cows were randomly selected and matched by parity and stage of lactation to create two virtually identical groups. Cows were also selected subject to their drying off date being later than the end of the study period, to avoid removal of the cows from the study group at dry off. During the 19-week study, twenty-one cows were removed from the study groups for veterinary intervention or due to being regrouped unexpectedly for drying off (Group 1: 9 cows, Group 2: 12 cows). Any cows that were removed from the study groups, remained absent for the remainder of the trial and were immediately replaced with an alternative cow (matched by parity and days in lactation), to maintain group size. Seventy-five of the originally selected cows remained present for the entirety of the trial (Group 1: 39 cows, Group 2: 36).

Group 1 consisted of 48 Holstein cows averaging (mean ± SD) 107.15 ± 57.42 days in milk (median: 106.50, IQR: 101.5, range: 25.00 – 232.00), producing on average 39.13 ± 10.78 L of milk/day, of parity 2.19 ± 1.21. The proportion of parity groups were parity 1: 0.38, parity 2: 0.25, parity 3: 0.25, parity 4+: 0.125. Group 2 consisted of 48 Holstein cows averaging 106.83 ± 56.79 days in milk (median: 102, IQR: 106.5, range: 26–215), producing on average 40.00 ± 10.67 L of milk/day, parity 2.19 ± 1.21. The proportion of parity groups were parity 1: 0.38, parity 2: 0.25, parity 3: 0.25, parity 4+: 0.13. Groups were moved into the study housing 1 week before the start of the trial for acclimatisation. The two groups of cows were managed simultaneously in adjacent pens within one building (Figure 1). Cows had been reared on the farm and all buildings on the farm housing adult cows had the same design as that of the experimental buildings used within the trial. Cows were managed in line with commercial care and management procedures at The University of Nottingham Center for Dairy Science Innovation.

### Treatment and experimental setup

**Figure 2:**
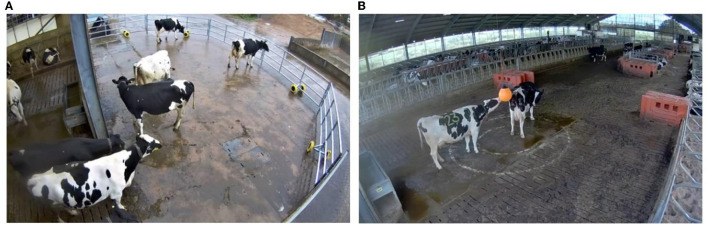
Images of the environmental resources provided as the intervention within the trial. **(A)** Displays the outdoor yard provided to Group 1. **(B)** Displays the indoor hanging novel object. Identical resources were provided to both groups of cows.

The intervention within this trial consisted of two different housing modifications to the standard living conditions of the cows. The first housing modification was the provision of a hanging novel object (inflated sailing buoy), suspended within an area of loafing space, situated at one end of the building (Figure 2). The specific novel object used was chosen because it had been deemed safe and practical in a preliminary study.

The second resource was access to an outdoor yard with a concrete floor (Figure 2). Both groups were provided with an identical outdoor yard. The yard boundaries were constructed from 5 mobile steel gates which were secured in place by interlocking chains between gates and drop bolts. The initial gate was fixed to the building wall whilst the other gate was secured to the access gate to the housing. The outdoor yards measured ~55 m^2^. The outdoor yards for Group 1 and Group 2 were situated opposite one another. Due to the close proximity, both yards provided almost identical outdoor views of the slurry collection area, an area of grassland used for storage and other farm buildings. A small covering of sand and grit was applied to the ground in icy weather conditions. Access to the outdoor yard was provided *via* an entry gate at the far end of the housing shed. All food, water and bedding areas were provided inside the building. During treatment periods when either one or both resources were made available, resources remained freely available 24 h a day for the entirety of that treatment period.

**Figure 3:**
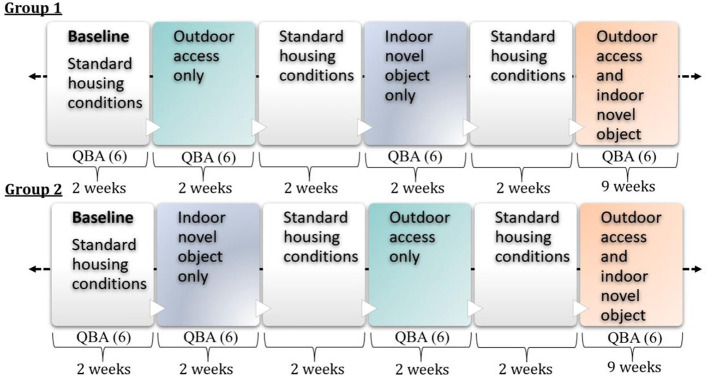
Timeline of the six consecutive treatment periods of the trial, displaying the length of time of each housing modification. Housing alterations were made on Mondays. The number of Qualitative Behavioural Assessments (QBA) that were conducted is displayed under each treatment period.

The trial ran for 19 weeks in total between the dates 22.11.2021 – 03.04.2022. The study timeline is illustrated in Figure 3. Groups were housed in standard housing conditions for 2 weeks to allow baseline observations to be taken. Standard housing conditions were as displayed in Figure 1, not including the outdoor yard or indoor novel object. Following this baseline period, both groups were given continuous 24-h access to a different enrichment resource for a period of 2 weeks. Group 1 were given access to the outdoor concrete yard and Group 2 were given access to a novel object within the building. Both resources were then removed and cows remained in standard housing conditions for 2 weeks. Following this period of standard housing, the initial treatment period was repeated but the resources were reversed, with Group 1 having access to the indoor novel object and Group 2 having access to an identical outdoor concrete yard. At the end of this two-week period, access to resources were removed and cows were housed in standard conditions for a further 2 weeks. Both groups of cows were then given continuous 24-h access to both resources for a period of 9 weeks.

### Quantification of enrichment use

Video footage was collected using 4 fixed Axis M1065 IP cameras. Use of the outdoor concrete yard and the novel object, were recorded throughout five separate, continuous 24-h periods. One 24-h period was sampled during the first treatment period, when Group 1 cows had access to the outdoor area and Group 2 cows had access to the indoor novel object. The corresponding 24-h period was sampled during the second treatment period when the resources had been switched, with Group 1 cows having access to the novel object and Group 2 cows having access to the outdoor concrete yard only. Three 24-h periods were sampled during the choice phase of the study. The first 24-h period was taken on the 05.01.2022 during the first week of the choice phase. The next two sampled 24-h periods were taken on 23.02.2022 (choice week 4) and 23.03.2022 (choice week 8). The 24-h periods were recorded from 00:00 to 24:00 and chosen to avoid veterinary or husbandry intervention with the cows.

Physical interaction with the novel object was classed as any physical contact of the object with any part of a cows body. Cow ID and length of interaction were recorded for every contact made with the object throughout all 24-h recording periods. Use of the outdoor yard commenced when a cow put one hoof over the entry line to the outdoor yard. A cows' time outside then ended the moment its entire body crossed over the entry line back into the building. Cow ID and time spent outside were recorded for every visit made outside throughout all 24-h recording periods.

### Qualitative behavioural assessment

One trained assessor completed one QBA for both groups of cows, three times per week, during every week of the trial (excluding non-treatment weeks when cows were in standard housing conditions). One QBA refers to one assessment, consisting of scoring the 20 descriptors, as outlined in the Welfare Quality Network protocol for dairy cows (36). The 20 terms used for every QBA were: active, relaxed, fearful, agitated, calm, content, indifferent, frustrated, friendly, bored, playful, positively occupied, lively, inquisitive, irritable, uneasy, sociable, apathetic, happy, distressed. The Assessments were made at the group level, which involved observation of all cows within the group. One QBA assessment was completed for both groups of cows, on Mondays, Wednesdays and Fridays, between 12:30 and 13:30. These days were chosen to avoid days where any form of human disturbance occurred, such as routine vet or foot trimming visits. The timeslot used to perform the QBA assessments was chosen to also avoid any routine management interference with the cows, such as feeding and cleaning. These days and times were therefore assumed to give the best indication of the herds undisturbed behaviour in these living conditions. The QBA assessment protocol and scoring sheet used was taken from the Welfare Quality Network Assessment Protocols for dairy cows (36) and was conducted by a trained assessor. The assessor observed the herd for 20 minutes in total, observing the expressive quality of group activity. If the cows were disturbed by the assessor's presence the assessment would be started a few minutes later when cows had resumed normal activity. This occurred infrequently due to the distance of the viewing platform from the living area of the cows. The assessor then moved away from the herd and scored the 20 descriptive terms manually on a visual analogue scale (VAS) on a paper form. Explanation of the VAS scoring system is provided by the Welfare Quality Network protocols (36). In brief, each VAS is defined by its left “minimum” and right “maximum” point, where minimum means the expressive quality indicated by the term is entirely absent in any of the animals and maximum means the expressive quality is dominant across all observed animals. It is possible to give more than one term a maximum score; animals could for example be both entirely calm and content. A score was then given for each term, by drawing a line on the assessment sheet on the visual analogue scale, at the point which best represented the level of that descriptive attribute to the herd. Each line point was manually measured in mm from the minimum mark to the given assessment line, resulting in a score between 0 and 125. Terms with positive connotations became more positive as the score increased and terms with negative connotations became more negative as the score became higher. To aid understanding of the terms used in the QBA assessment for dairy cattle from the Welfare Quality Assessment Protocols (36), definitions for each descriptor were checked *via* the Cambridge Dictionary online (50). The QBA assessor spent 2 weeks conducting QBA assessments on cows housed in the experimental buildings as part of training.

### Weather

The temperature (°C) throughout the trial was recorded using an “Imonnit” weather sensor (Monnit Corporation, Utah, US). The sensor took a temperature recording every 2 h, throughout 24-h, providing 12 data points per day. The sensor was secured to the outside of the building, within the outdoor yard for Group 2. Given the close proximity of the outdoor yards, this sensor was accepted to provide weather details for the overall outdoor area used by both groups of cows.

### Statistical analysis

All statistical analyses were performed using packages readr (51), dplyr (52), tidyverse (53), stats (54) and FactoExtra (55) in RStudio version 4.1.2 (56). The raw QBA linear measurements were centred and standardised to create a normal distribution for further analysis. QBA data were analysed using a principal component analysis (PCA), a multivariate technique of particular value to assess data consisting of correlated quantitative dependent variables. The procedure leads to the production of “principal components”; new variables which summarise information from the correlated variables (57). Descriptive assessment of QBA was conducted graphically to facilitate visualisation of the important variables contributing to the key principal components. The first two principal components, explaining the highest percentage of the variance of the data and with eigen values >1.0, were used for additional inferential analysis in line with standard procedure (57). A conventional linear model was constructed to test the effect of treatment period on principal component scores. Explanatory variables were retained in the models when *P* < 0.05. QBA results were evaluated separately for Group 1 and Group 2 – as well as in combination. Quantity of QBA assessments was matched, with 6 QBA assessments per group per treatment period. To achieve 6 QBA assessments for the final treatment period which lasted 9 weeks rather than the 2 weeks of other periods, we performed 3 QBAs in the first 2 weeks of this period and another 3 in the last 2 weeks.

Quantification of enrichment use is reported as the mean (±SD) time per cow spent using enrichment resources per treatment period. Treatment period refers to the single continuous 24-h period of footage from which results were obtained. Results are reported as the mean ± standard deviation. The percentage of the group that used the resource refers to the percentage of cows that used it during a specific 24-h period.

## Results

### Quantification of enrichment use

When cows were provided access to the indoor novel object only, they spent 6.34 ± 4.62 (Group 1) and 10.13 ± 8.66 (Group 2) min per day interacting with it. The percentage of the group that used the novel object was 94.87% (Group 1) and 100% (Group 2). When cows were provided with access to the outdoor yard only, they spent 55.67 ± 32.11 (Group 1) and 102.26 ± 59.92 (Group 2) min per day outside. The percentage of the group that used the outdoor yard was 94.87% (Group 1) and 100% (Group 2).

During the early choice phase, when cows had simultaneous access to both resources, Group 1 cows spent 4.91 ± 5.41 min per day using the indoor novel object and 98.37 ± 57.57 min per day outside. The percentage of Group 1 using the indoor novel object during this period was 94.87% compared to 97.44% using the outdoor yard. During the early choice phase, Group 2 cows spent 9.6 ± 7.58 min per day using the indoor novel object and 114.38 ± 55.28 min per day using the outdoor yard. The percentage of Group 2 using the indoor novel object during this phase was 94.44% compared to 97.22% using the outdoor yard.

During the late choice phase, Group 1 cows spent 3.12 ± 3.27 min per day using the indoor novel object and 55.06 ± 31.32 min per day using the outdoor yard. The percentage of Group 1 using the resources during this phase was 87.18% (indoor novel object) and 94.87% (outdoor yard). During the late choice phase, Group 2 cows spent 2.3 ± 2.66 min per day using the indoor novel object and 91.46 ± 47.02 min per day using the outdoor yard. The percentage of the Group 2 using the resources during this phase was 86.11% (indoor novel object) and 97.22% (outdoor yard).

### QBA results Group 1

**Figure 4:**
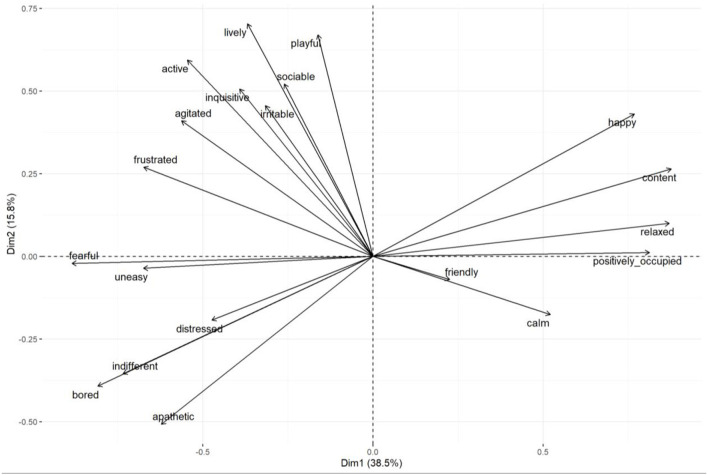
Group 1: Variable correlation plot displaying the relationship between all variables in PC1 (Dim1) and PC2 (Dim2) and each terms value figure in contribution to the principal component.

PCA of the QBA scores for Group 1 cows identified 5 principal components with eigen values >1. However, the first two principal components explained the majority of the variance in the data and were therefore retained for analysis. The first principal component (PC1) accounted for 38.45% of the variance and displayed the most positive correlating adjectives of “content”/“relaxed”, with the most negative correlating adjectives of “fearful/bored”. The second component (PC2) explained 15.76% of the variance and comprised of the most positive correlating adjectives of “lively/playful” and the most negative correlating adjectives of “apathetic/bored”. Table 1 displays the full list of adjectives for both components with associated correlation value. Figure 4 displays the relationship between all variables in PC1 and PC2.

**Table 1:** Group 1: Principal components 1 and 2, displaying associated correlations between variables and principal components for each behavioural descriptor.

**Descriptor**	**PC1**	**PC2**
Active	−0.54	0.59
Relaxed	**0.87**	0.10
Fearful	–**0.88**	−0.02
Agitated	−0.56	0.41
Calm	0.52	−0.18
Content	**0.88**	0.26
Indifferent	−0.73	−0.36
Frustrated	−0.67	0.27
Friendly	0.23	−0.07
Bored	–**0.81**	–**0.39**
Playful	−0.16	**0.67**
Positively occupied	0.81	0.01
Lively	−0.37	**0.70**
Inquisitive	−0.39	0.51
Irritable	−0.32	0.46
Uneasy	−0.67	−0.04
Sociable	−0.26	0.52
Apathetic	−0.62	–**0.51**
Happy	0.77	0.43
Distressed	−0.47	−0.19

The bold values indicate the two most positive and two most negative correlating adjectives.

**Figure 5:**
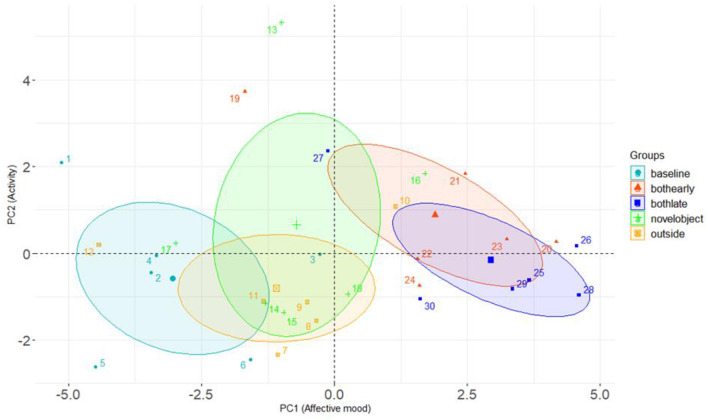
Group 1: Biplot displaying all QBA assessment scores in terms of PC1 and PC2. Each separate point displays one assessment date. Points are coded per treatment period [baseline, novel object, outside, both resources (early) and both (late)] as indicated on the plot. Group means are in bold and ellipses indicate the 95% confidence intervals of the group mean.

Results of the mixed effect linear model for cows in Group 1 are presented in Table 2. Treatment period had a significant effect on PC1, with cows scoring higher values on this component during the choice period (when cows had access to both the outdoor yard and the novel object, both at the beginning and at the end of this phase) compared to the baseline weeks. Cows also scored significantly higher on this component when they solely had access to the indoor novel object, compared to the baseline week. Higher scores on PC1 reflected cows being assessed as more content, relaxed and positively occupied compared to fearful, bored and indifferent. The effect of treatment period on PC2 was non-significant. The difference in PC1 and PC2 between treatment period are presented graphically in Figure 5.

**Table 2:** Group 1: Results of the linear model assessing PC1 and PC2 scores attained during the different treatment periods.

**Coefficients of the model**	**Estimate**	**Confidence interval (95%)**	**P-value**
Reference: Baseline housing conditions Intercept	−3.04		
Choice phase (early)	4.94	2.7–7.11	<0.01
Choice phase (late)	6.00	3.82–8.17	<0.01
Indoor novel object	2.33	0.16–4.51	0.04
Outside	1.95	−0.22–4.13	0.07

Choice phase (both resources simultaneously available), indoor novel object only and outside (access to the outdoor yard only).

### QBA results Group 2

**Figure 6:**
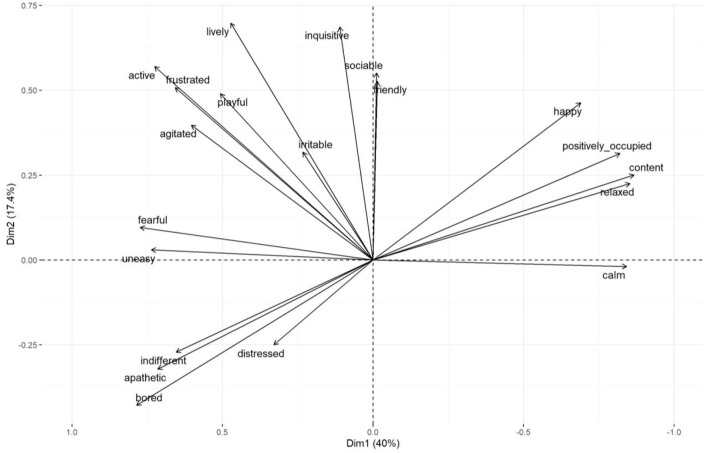
Group 2: Variable correlation plot displaying the relationship between all variables in PC1 (Dim1) and PC2 (Dim2) and each terms value figure in contribution to the principal component.

PCA of the QBA scores for Group 2 cows identified 5 principal components with eigen values >1. The first two principal components explained the majority of the variance in the data and were therefore retained for analysis. The first principal component (PC1) accounted for 40.03% of the variance and displayed the most positive correlating adjectives of “bored/fearful”, with the most negative correlating adjectives of “content/relaxed”. The second component (PC2) explained 17.39% of the variance and comprised of the most positive correlating adjectives of “lively/inquisitive” and most negative correlating adjectives of “bored/apathetic”. Table 3 displays the full list of adjectives for both components with associated correlation value. Figure 6 displays the relationship between all variables in PC1 and PC2.

**Table 3:** Group 2: Principal components 1 and 2, displaying associated correlations between variables and principal components for each behavioural descriptor.

**Descriptor**	**PC1**	**PC2**
Active	0.73	0.57
Relaxed	–**0.85**	0.22
Fearful	**0.77**	0.10
Agitated	0.60	0.40
Calm	−0.84	−0.02
Content	–**0.87**	0.25
Indifferent	0.65	−0.27
Frustrated	0.66	0.51
Friendly	−0.01	0.53
Bored	**0.79**	–**0.43**
Playful	0.51	0.49
Positively occupied	−0.82	0.31
Lively	0.47	**0.70**
Inquisitive	0.11	**0.69**
Irritable	0.23	0.32
Uneasy	0.74	0.03
Sociable	−0.01	0.55
Apathetic	0.71	–**0.32**
Happy	−0.69	0.46
Distressed	0.33	−0.25

The bold values indicate the two most positive and two most negative correlating adjectives.

**Figure 7:**
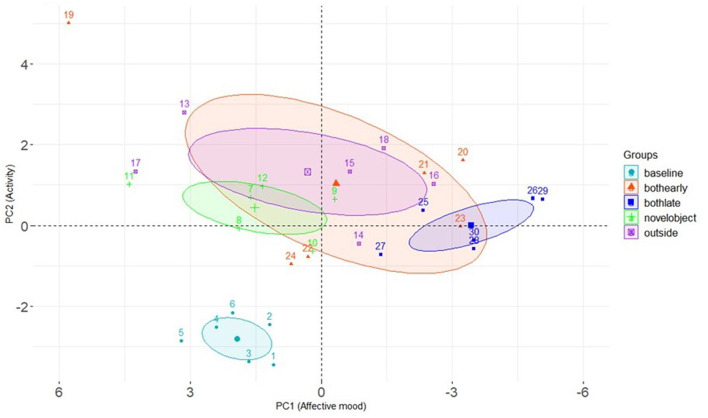
Group 2: Biplot displaying all QBA assessment scores in terms of PC1 and PC2. Each separate point displays one assessment date. Points are coded per treatment period [baseline, novel object, outside, both resources (early) and both (late)] as indicated on the plot. Group means are in bold and ellipses indicate the 95% confidence intervals of the group mean.

Results of the mixed effect linear model for cows in Group 2 are presented in Table 4. Treatment period had a significant effect on PC1, with cows scoring lower values on this component during the late stage of the choice period (when cows had access to both the outdoor yard and the novel object) compared to the baseline weeks. Lower scores on PC1 reflected cows being assessed as more content, calm and relaxed, compared to bored and fearful. Treatment period also had an effect on PC2, with cows scoring higher values on this component during all treatment periods compared to baseline. Higher scores on PC2 reflected cows being assessed as more lively, inquisitive and active compared to bored, apathetic and indifferent. The difference in PC1 and PC2 between treatment periods are presented graphically in Figure 7.

**Table 4:** Group 2: Results of the linear model assessing PC1 and PC2 scores attained during the different treatment periods.

**Coefficients of the model**	**Estimate**	**Confidence interval (95%)**	**P-value**
**QBA PC1**			
Reference: Baseline housing conditions Intercept	1.93		
Choice phase (early)	−2.27	−4.91–0.38	0.09
Choice phase (late)	−5.37	−8.01 to −2.72	<0.01
Indoor novel object	−0.41	−3.05–2.24	0.76
Outside	−1.62	−4.26–1.03	0.22
**QBA PC2**			
Intercept	−2.80		
Choice phase (early)	3.83	2.40–5.25	<0.01
Choice phase (late)	2.80	1.38–4.22	<0.01
Indoor novel object	3.24	1.81–4.66	<0.01
Outside	4.13	2.71–5.55	<0.01

Choice phase (both resources simultaneously available), indoor novel object only and outside (access to the outdoor yard only).

### QBA results Group 1 and 2 combined

**Figure 8:**
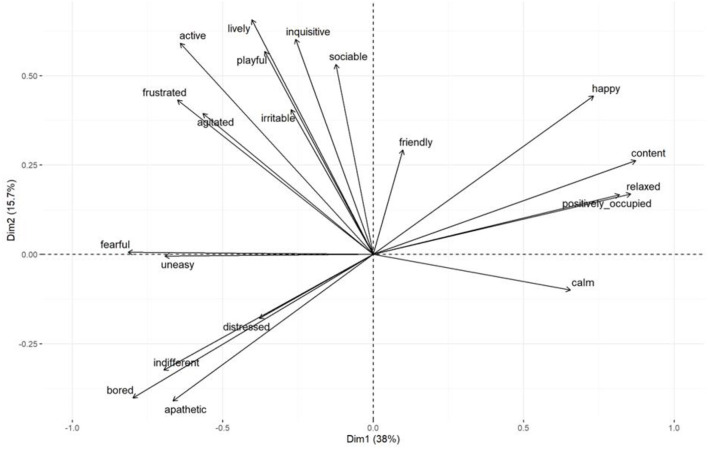
Group 1 and 2 combined: Variable correlation plot displaying the relationship between all variables in PC1 (Dim1) and PC2 (Dim2) and each terms value figure in contribution to the principal component.

PCA of the QBA scores for Group 1 and 2 combined identified 5 principal components with eigen values >1. The first two principal components explained the majority of the variance in the data and were therefore retained for analysis. The first principal component (PC1) accounted for 37.96% of the variance and displayed the most positive correlating adjectives of “content/relaxed”, with the most negative correlating adjectives of “fearful/bored”. The second component (PC2) explained 15.67% of the variance and comprised of the most positive correlating adjectives of “lively/inquisitive” and most negative correlating adjectives of “apathetic/bored”. Table 5 displays the full list of adjectives for both components with associated correlation value. Figure 8 displays the relationship between all variables in PC1 and PC2.

**Table 5:** Group 1 and Group 2: Principal components 1 and 2, displaying associated correlations between variables and principal components for each behavioural descriptor.

**Descriptor**	**PC1**	**PC2**
Active	−0.64	0.59
Relaxed	**0.86**	0.17
Fearful	**−0.81**	0.01
Agitated	−0.57	0.39
Calm	0.66	−0.10
Content	**0.87**	0.26
Indifferent	−0.70	−0.32
Frustrated	−0.65	0.43
Friendly	0.10	0.29
Bored	**−0.80**	**−0.40**
Playful	−0.36	0.57
Positively occupied	0.82	0.17
Lively	−0.40	**0.66**
Inquisitive	−0.26	**0.60**
Irritable	−0.27	0.40
Uneasy	−0.69	−0.004
Sociable	−0.12	0.53
Apathetic	−0.67	**−0.41**
Happy	0.73	0.44
Distressed	−0.38	−0.18

The bold values indicate the two most positive and two most negative correlating adjectives.

**Figure 9:**
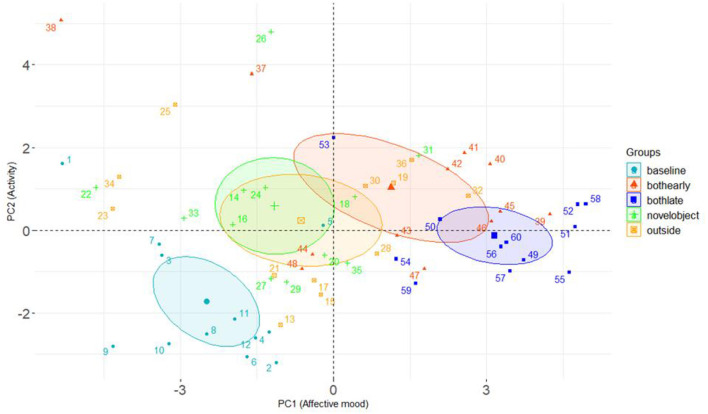
Group 1 and 2 combined: Biplot displaying all QBA assessment scores in terms of PC1 and PC2. Each separate point displays one assessment date. Points are coded per treatment period [baseline, novel object, outside, both resources (early) and both (late)] as indicated on the plot. Group means are in bold and ellipses indicate the 95% confidence intervals of the group mean.

Results of the mixed effect linear model for the combined results for Group 1 and Group 2 cows are presented in Table 6. Treatment period had a significant effect on PC1, with cows scoring higher values on this component during the choice period (when cows had access to both the outdoor yard and the novel object, both at the beginning and at the end of this phase) compared to the baseline weeks. Cows also scored significantly higher on this component when they solely had access to the outdoor yard, compared to the baseline week. Higher scores on PC1 reflected cows being assessed as more content, relaxed and positively occupied compared to fearful and bored. Treatment period also had a significant effect on PC2, with cows scoring higher values across all treatment periods compared to baseline weeks. Higher scores on PC2 were indicative of cows being assessed as more lively and inquisitive compared to apathetic and bored. The difference in PC1 and PC2 between treatment period are presented graphically in Figure 9.

**Table 6:** Group 1 and 2 combined: Results of the linear model assessing PC1 and PC2 scores attained during the different treatment periods.

**Coefficients of the model**	**Estimate**	**Confidence interval (95%)**	**P-value**
**QBA PC1**			
Reference: Baseline housing conditions Intercept	−2.49		
Choice phase (early)	3.62	1.99–5.25	<0.01
Choice phase (late)	5.65	4.02–7.27	<0.01
Indoor novel object	1.34	−0.29–2.96	0.11
Outside	1.85	0.22–3.48	0.03
**QBA PC2**			
Intercept	−1.72		
Choice phase (early)	2.74	1.48–4.01	<0.01
Choice phase (late)	1.59	0.33–2.86	<0.01
Indoor novel object	2.31	1.04–3.58	<0.01
Outside	1.97	0.70–3.23	<0.01

Choice phase (both resources simultaneously available), indoor novel object only and outside (access to the outdoor yard only).

An overview of the study results are presented in Table 7.

**Table 7:** Summary of results displaying PC1 and PC2 for each group and their associated most positive and negative correlating adjectives.

	**Principal component**	**Treatment period**
Group 1	PC1 **content/relaxed** – bored/fearful	**Indoor novel object** only Outside only **Choice phase (early)** **Choice phase (late)**
	PC2 **lively/playful** – bored/apathetic	Indoor novel object only Outside only Choice phase (early) Choice phase (late)
Group 2	PC1 **content/relaxed** – fearful/bored	Indoor novel object only Outside only Choice phase (early) **Choice phase (late)**
	PC2 **lively/inquisitive** – bored/apathetic	**Indoor novel object only** **Outside only** **Choice phase (early)** **Choice phase (late)**
Combined	PC1 **content/relaxed** – bored/fearful	Indoor novel object only **Outside only** **Choice phase (early)** **Choice phase (late)**
	PC2 **lively/inquisitive** – apathetic/bored	**Indoor novel object only** **Outside only** **Choice phase (early)** **Choice phase (late)**

Treatment periods which had a significant effect on principal components are indicated for each group. Treatment periods in bold indicate where significantly higher scores were being attained on a principal component. Adjectives in bold are the most positively correlating terms for that principal component.

### Weather

The mean (±SD) air temperature (°C) throughout treatment periods were: 3.62 + 4.10 (baseline), 6.46 + 3.74 (Group 1 outdoor yard, Group 2 indoor novel object), 2.73 + 4.27 (Group 1 indoor novel object, Group 2 outdoor yard), 7.05 + 3.26 (early choice period) and 6.36 + 6.67 (late choice period).

## Discussion

### Summary

The current study is the first to utilise QBA to assess dairy cows' affective states in response to a potentially positive welfare intervention. The intervention consisted of manipulating the standard housing conditions of commercially housed dairy cows. Diversification of the environment through environmental enrichment, which offers opportunities for exploration, control and choice, has been suggested as one way to offer confined animals positive experiences (12). In line with this theory, the hypothesis of the study was that offering additional environmental resources would have a positive impact on cows' affective states. The results support this hypothesis, with more positive affective states being indicated during intervention periods, when cows had access to additional environmental resources.

### PC1 (affective mood) and treatment period

Group 1 cows scored significantly higher on PC1 during both stages of the choice period, when cows had access to both resources and also when they just had access to the indoor novel object compared to standard housing conditions. Similar results were shown for Group 2 cows, however only during the late choice phase compared to baseline conditions. Combined results from both groups showed that cows scored higher on PC1 during both stages of the choice phase and when cows had access to the outdoor yard compared to standard housing conditions. The most positively correlated adjectives on PC1 were relaxed, content and positively occupied, all with positive emotional connotations. The most negatively correlating terms were fearful and bored, therefore this principal component could be representative of general affective mood, on a scale from negative (lower scores) to positive (higher scores).

These results suggest that when cows had access to additional environmental resources, they were more relaxed, content and positively occupied than when in standard housing conditions. Although the behavioural descriptors were not analysed in isolation, reference should be made to why cows may have scored higher on these terms during treatment periods. Positively occupied is arguably the least complicated term to interpret. It seems plausible that increasing the behavioural activities available within the cows' environment would increase the amount of time they spent positively occupied. This has previously been observed in calves, where the provision of four different types of enrichment simultaneously resulted in calves spending more time interacting with enrichment compared to when only one single item was provided (58). Similarly in pigs, the simple provision of four instead of two wooden beams, increases both the frequency and duration of manipulation bouts (59) and increasing the amount of straw available increased both the time spent manipulating the straw and pigs' simultaneous straw use (60).

Cows appearing more relaxed and content would be in line with an overall shift to a more positive affective mood. It is possible that as animals spend more time positively occupied with the environment, they use more positively motivated energy, which could be linked to being more relaxed/tired out. Dairy cows provided with overnight pasture access have been shown to have longer overnight lying durations compared to continually housed cows (61). Longer durations of sleep behaviour have been observed in rats provided with environmental enrichment, compared to rats housed in standard cages (62). Furthermore, rats housed in more complex environments with a choice of simultaneously available enrichment resources compared to having only one type of enrichment, have been shown to sleep more, spend more time in enrichment-directed behaviour and less time inactive while awake (63, 64).

The results from PC1 also suggest that cows were less bored and fearful when they had access to additional environmental resources. Research on boredom in animals suggests that it may be reduced by providing additional behavioural opportunities through environmental enrichment (65–67). It therefore seems biologically plausible for cows to have appeared less bored when they were provided with two additional environmental resources. Understanding why a simple change in environmental conditions may reduce wider negative affective states, such as apathy and fear however, is more challenging. In human psychology, the experience of boredom is described as unpleasant and distressing (68). Given that this is an under-researched area in animals (69), it is possible it is an equally aversive experience and research has started to suggest this, for example animals will choose aversive experiences over monotony (65, 70). In animals, depression-like symptoms appear to be induced by barren housing, which may develop from unavoidable chronic stressors of the environment (71). Proxies of low mood, one symptom of depression, such as negative information processing, have shown to be changed in pigs, but not dairy cows, through environmental enrichment (19, 72). When pigs were moved from barren to enriched housing, they showed decreased negative information processing in cognitive bias tests. This result was exemplified in pigs that were transferred from enriched to barren housing, showing higher levels of negative information processing in cognitive bias tests when compared to that of pigs that had always been managed in barren housing (19). Dairy cows were housed in conditions aimed to elicit a contrast in positive and negative affective states (72). The ‘positive' housing provided additional space, enrichment and social stability, with a ‘negative' condition featuring overcrowding, removal of enrichment and social instability. The contrasting housing conditions however, failed to influence responses to a judgement bias test. Further research is needed to understand the impact that housing conditions have on both positive and negative affective states in animals.

Boredom is correlated with anxiety and fear in people (73, 74), and therefore it is possible that these negative affective states are also linked in animals. Given the association between these states, reducing boredom could simply be paired with overall reductions in negative affective states such as fear, or providing more time filling environmental activities could act as some form of distraction from triggers of fear and anxiety. Increasing animals' time in positively engaging behaviours would likely decrease time spent in empty or boredom-like situations, where cows may be more aware of surroundings and potential threats. Interestingly, anxiety behaviours in rats and mice have shown to be reduced through the use of environmental enrichment (75–77) but this link has yet to be explored in dairy cows.

The potential benefits in behaviour and welfare of dairy cows facilitated by different forms of environmental enrichment has started to be explored (29). However, the impact of enrichment on cows' affective wellbeing has received little research. Results indicating enhanced affective states have been shown in calves housed in pairs compared to individually, and in calves housed in enriched compared to unenriched environments (15, 16). A small number of studies have explored the association between the level of housing confinement and affective states in dairy cows. Reduced reward anticipation has been displayed in dairy cows with access to pasture (25), however mixed results have been shown when evaluating eye temperature between these conditions, a physiological indicator of stress (78). QBA has indicated better affective states in dairy cows in loose housing systems compared to tethered systems and during the early stage of a housing period compared to during the late stage of housing (13, 14). The results of the current study, indicating more positive affective states in cows with outdoor access, appear consistent with these findings. The significant results from PC1 which are suggestive of an overall shift to a more positive mood, including cows appearing less bored, persisted during the late stage of the choice period, when cows appeared to have started to show some level of habituation to the indoor novel object. However, despite some decline in time spent using one of the enrichments, the majority of both groups were still interacting with it. Although further replication of the current study would be beneficial, the similarity between results for the two groups of cows shows the repeatability of the study findings.

### PC2 (activity) and treatment period

Results from Group 2 and both groups combined, showed that cows scored significantly higher on PC2, during all treatment periods compared to standard housing conditions. The most positive correlating adjectives on this component were lively and inquisitive, with the most negative correlating terms being apathetic and bored. This component therefore, appears to represent a combination of activity and valence. Enrichment is known to increase exploration and associated activity (79, 80). Research in calves has shown that simple housing modifications, such as social housing and additional space are associated with higher levels of play behaviour (81, 82). Dairy cows and heifers have also been shown to display increased activity and play behaviour with decreased access to exercise (83, 84) suggesting a motivational need for locomotory behaviour which is limited during confinement. Therefore, it would be understandable for cows to appear more active, lively or inquisitive when provided with enrichment resources. Furthermore, the provision of access to outside space was likely facilitative to increased exercise in the current study. Again, when compared to the field of human psychology, exercise is a known and widely used treatment for anxiety and depression (85, 86) and it could be possible for a similar relationship to exist in animals. A review on the literature on the benefits of exercise for dairy cows has confirmed that increasing the movement opportunity provided by housing has a positive effect on activity level and can benefit cow health, behaviour and welfare (87). The provision of diverse environments offering wider behavioural activities is already suggested to be one of the first strategies for mitigating boredom in confined animals (67). Inactivity, one suggested behavioural expression of boredom in animals (88) has been shown to decrease in multiple species when provided with more complex environmental opportunities (65, 89, 90). Concurrent with the knowledge of the relationship between environmental enrichment and boredom, it is tenable that the cows in the current study displayed a decreased behavioural expression of boredom when provided with access to two additional environmental resources.

The temperature throughout the study remained well within the thermal comfort zone for dairy cows (91, 92). In addition to this, the temperature between treatment periods varied within a small range of 4.32°C, therefore was unlikely to have impacted cow behaviour or affective states.

### QBA and study limitations

QBA is utilised for its on-farm practicality, requiring little time to complete when compared to other farm assurance assessments (45), and requiring no resources or technical equipment. Its practicality as an on-farm measure of welfare assessment, including aspects of positive welfare, was observed within the present study however it should be mentioned that some terms are more challenging to assess than others. Although QBA does not assess the physical behaviours performed by the animal, the particular behaviour an animal is engaged in can affect the ease with which the expressive qualities of that behaviour are assessed. Behaviour may therefore carry more weight in our interpretation of animals' quality of experience for some terms compared to others. For example, a cow that is positively occupied could be observed and its style of behaviour could be assessed for varying terms, such as how tense or relaxed it may appear. However, a cow standing or lying completely motion less, is more challenging to assess for its level of happiness or frustration for example. The expressive qualities of animals therefore appear to be more difficult to assess when less active, with fewer visual cues. Overall terms were assessable, yet a small number were much more challenging to assess, the term happy being one such example. Although the concept of QBA is to use interpretation of animals' expressive demeanour to make the assessments, a certain level of knowledge as to how these affective descriptions may be behaviourally expressed within a certain species is needed for guidance. Very little is known about how animals express happiness, therefore making a visual judgement of an animal's level of happiness is a challenging task, which ties in to the current complexities of trying to evaluate animals' affective experiences (10). QBA has proven itself as a reliable measure of making inferences about animals' differing affective states (10), yet the potential for it to be considered as anthropomorphic is frequently mentioned (30, 31, 93). This criticism could potentially be controlled, by using careful consideration of the terms used for assessments or by also using the free choice profiling approach, where assessors generate their own terms.

A potential limitation of the QBA assessments conducted within this study was the inability to blind the assessor to study treatments. Therefore, the assessor was aware of when cows were housed in standard and enriched conditions. It is possible that this could introduce an element of unconscious assessor bias, due to interventions having the potential to be linked to moral connotations, for example one treatment being perceived as better for welfare than another. Evidence of this contextual bias has previously been observed whilst using QBA (94, 95). Tuyttens et al. (94) recruited veterinary students to assess the welfare of laying hens, using QBA from video recordings. The same video clip from one group of hens was split into two separate clips and students were informed that one showed hens from an organic farm, with the other showing hens from a conventional farm. Students gave lower scores for negative descriptors and higher scores for positive descriptors when under the impression that the hens they were scoring were from an organic rather than a non-organic farm. The magnitude of this relationship was positively correlated with students opinion regarding hen welfare in these different systems. Wemelsfelder et al. (96) investigated the impact of being contextually aware of the animals' environment on QBA results. Video recordings of 15 pigs interacting with a novel object were digitally extracted and applied to both an indoor and outdoor setting and the resultant video clips were analysed by blind observers. There was a strong correlation between the indoor and outdoor variants of video clips across both QBA components. Environmental background did however have an effect on one of the QBA components (confident/content–cautious/nervous) but not the other (playful/active–bored/lethargic), implying that pigs observed in an outdoor setting were perceived to be more confident and content and less cautious and nervous than when these same pigs were observed against an indoor background. Thus, although different contexts led to slight shifts in assessors' scorings in this study this did not lead to significant misinterpretations of the pigs body language. One of the underpinning concepts of QBA is to evaluate not just how animals are behaving, but how they are interacting with their environment (30, 97), which evidently also requires knowledge of the environmental situation. Therefore, the assessor's use of knowledge regarding the animal's environment does not seem unreasonable. This contextual bias is a potential weakness of the use of QBA, when used in certain situations, where different farming systems, conditions or study interventions may be perceived to provide different levels of welfare. Ideally observers should be blind to such background conditions, and in many QBA studies they are (41), however in live assessments of changing on-farm housing conditions that is not possible to achieve. QBA has been practically implemented in industry as an on-farm welfare assessment (36, 37) despite this potential bias risk, due to contextual awareness of surroundings.

Results of the current study could have been strengthened through use of a combination of positive welfare indicators. For example, correlates of enhanced affective states have been demonstrated between QBA results and ear position in calves and lambs (98, 99) and QBA and positive social behaviour in dairy cows (46). Divergences may also highlight where studies making reference to changes in affective states require further replication or validation. For example, Carreras et al. (20) evaluated the affective states of pigs housed in an enriched (solid floor, straw and increased space allowance) or a barren (decreased space allowance, no straw, slatted floors) environment and found QBA results, cortisol concentrations and carcass wounds to be indicative of better welfare states in the enriched conditions. However, no differences were detected in the cognitive bias testing. Similarly, Vitali et al. (100) evaluated the welfare status of pigs housed in mechanically compared to naturally ventilated housing and found QBA to identify pigs in mechanically ventilated buildings to be associated with more positive affective states. Interestingly, pigs in mechanically ventilated buildings also performed higher levels of stereotypical and negative social behaviours and showed a higher general level of inactivity, all behaviours associated with negative affective states (88, 101, 102).

## Conclusions

Qualitative behavioural assessment was used to identify differences in cow behavioural expression and, we therefore hypothesise, in associated affective state, between periods when the cows were housed in standard commercial conditions and periods when they were housed in enriched conditions. The enriched conditions provided additional environmental resources. Our results indicate that the simple housing modifications, access to a novel object and to outdoor space, are likely to positively impact the affective lives of commercially housed dairy cows. The results are biologically plausible and suggest that some level of positive experience may be facilitated through simple modification to the housed environment of dairy cows.

## Data availability statement

The raw data supporting the conclusions of this article will be made available by the authors, without undue reservation.

## Ethics statement

The animal study was reviewed and approved by the University of Nottingham, School of Veterinary Medicine, and Science Ethical Review Committee.

## Author contributions

AR contributed to study conception and design, on farm management of the study including data collection, data analysis, interpretation of data, and writing of the manuscript. MG contributed to study conception and design, data analysis, interpretation of data, and reviewing the manuscript. LR, JK, and NE contributed to study conception, design, and reviewing the manuscript. All authors contributed to the article and approved the submitted version.
